# Activated PI3 Kinase Delta Syndrome: Molecular Pathogenesis and Emerging Therapeutics

**DOI:** 10.7759/cureus.90448

**Published:** 2025-08-18

**Authors:** Elias A Alraqibah

**Affiliations:** 1 Department of Medicine, College of Medicine, Qassim University, Buraydah, SAU

**Keywords:** activated pi3 kinase delta syndrome (apds), immune dysregulation, phosphoinositide 3-kinase, pi3kδ pathway, primary immunodeficiency

## Abstract

Activated PI3 kinase delta syndrome (APDS) is a rare, inherited primary immunodeficiency characterized by gain-of-function mutations in the *PIK3CD* or *PIK3R1* genes, resulting in hyperactivation of the PI3Kδ pathway and consequent immune dysregulation. This review provides an in-depth exploration of the genetic and molecular mechanisms underlying APDS, highlighting the complex interplay between immunodeficiency and autoimmunity. Clinical manifestations include recurrent infections, lymphoproliferation, autoimmune cytopenias, and inflammatory complications, which often begin in early childhood. Diagnostic strategies have evolved with the advent of genetic sequencing and immunologic biomarkers, enabling the more accurate identification and classification of APDS subtypes than previously possible. Therapeutic advances, particularly the development of PI3Kδ inhibitors such as leniolisib and duvelisib, have significantly improved patient outcomes by targeting the underlying molecular defects. Additional management approaches include immunoglobulin replacement, immunomodulators, and hematopoietic stem cell transplantation in severe cases. Despite these advances, challenges such as delayed diagnosis, treatment-related complications, and variability in clinical presentation persist. Continued research on targeted therapies, long-term outcomes, and gene-editing technologies is essential to optimize care and enhance the quality of life for individuals with APDS.

## Introduction and background

The phosphoinositide 3-kinase (PI3K) pathway is an important signaling cascade that regulates a wide array of cellular processes, including cell growth, proliferation, survival, metabolism, and immune function [1,2]. PI3K has different isoforms, and the delta isoform (PI3Kδ) is an essential component that significantly regulates the immune system, primarily expressed in hematopoietic cells [3,4]. PI3Kδ is important for activating and developing different immune components, including T and B cells, and plays a significant role in the response to antigen-receptor signaling [5]. This pathway also influences cytokine production and cellular trafficking in the lymphoid tissues [5]. Dysregulation or dysfunction of this pathway is associated with the incidence of different immune-related disorders, such as hyperactivation of immune responses, primary immunodeficiency syndrome, and autoimmunity [6,7]. In this context, gain-of-function mutations in the PI3Kδ subunit have been implicated in a rare but significant immunological disorder known as activated PI3 kinase delta syndrome (APDS) [8,9]. Understanding this pathway is crucial for grasping the various mechanisms related to the occurrence of APDS and plays a significant role in exploring and developing targeted therapeutic interventions for APDS.

APDS, first described in 2013, marked a significant advancement in the study of primary immune deficiency. This condition occurs because of gain-of-function mutations in the *PIK3CD* or *PIK3R1* genes, which encode the catalytic (*p110δ*) and regulatory (*p85α*) subunits of the delta isoform, respectively, resulting in immune dysfunction [10,11]. Hyperactivation of the PI3Kδ pathway occurs following these mutations, leading to a series of pathological consequences, including lymphoproliferation, repeated infections, and immune dysregulation [12]. Based on the genetic mutations involved, APDS is currently divided into two primary subtypes: mutations in *PIK3CD* cause APDS1 and mutations in *PIK3R1* result in APDS2 [13]. Although the two subtypes exhibit many similar symptoms, precise genetic testing is necessary to confirm the diagnosis, as subtle variations in presentation and genetic mechanisms have been observed [8]. Emphasizing the need for genetic and molecular definitions to understand immune system concerns, APDS has become a vital field of study since its discovery.

Significant advances in the diagnosis and management of APDS have been reported recently. Earlier and more precise diagnoses have resulted from advances in genomic technologies, which have helped identify genetic mutations correlated with the syndrome [8]. In addition, the treatment of patients with APDS has now shifted to the use of targeted treatments with PI3Kδ inhibitors that aim to improve outcomes and increase the expected quality of life (QoL) of affected patients [13]. However, there are many remaining barriers to the complete management of these conditions, including delays in the diagnosis of the disorders, variations in the symptoms between patients, and possible long-term side effects of the proposed treatment. Therefore, it is vital to maintain current knowledge of APDS to bridge these gaps and improve patient care [8]. The current review aims to understand the genetics, mechanisms, clinical characteristics, diagnosis, and development of possible treatments for APDS based on a summary of the most recent studies on APDS. This overview emphasizes the development achieved and maps out the next steps for study and clinical practice in this rapidly changing field by compiling recent research.

## Review

Methodology

This narrative review was conducted using a selective literature search strategy. We searched PubMed, Google Scholar, and ScienceDirect databases for peer-reviewed articles published between 2010 and 2024 using the following keywords: "Activated PI3K Delta Syndrome", "APDS", "PIK3CD mutation", "PI3Kδ inhibitors", "leniolisib", and "duvelisib". Articles were included if they discussed the pathophysiology, clinical features, diagnosis, or management of APDS. Case reports, review articles, and clinical trials were all considered. Studies were excluded if they focused solely on unrelated PI3K isoforms or non-APDS immunodeficiencies. No formal PRISMA (Preferred Reporting Items for Systematic Reviews and Meta-Analyses) guidelines were followed, given the narrative nature of the review, though care was taken to include diverse and recent sources.

Genetics and molecular pathophysiology

Mutations in the PIK3CD and PIK3R1 Genes

Mutations in the *PIK3CD* and *PIK3R1* genes lead to a gain-of-function in APDS, a monogenic condition, as they encode crucial elements of the PI3Kδ pathway. PIK3CD encodes the catalytic subunit of PI3Kδ, *p110δ*, and PIK3R1 encodes the regulatory subunit *p85α* [6]. Mutations in these genes lead to hyperactivity of the PI3Kδ signaling pathway, which interferes with regular immune activity [14]. Mutations in *PIK3CD*, found in APDS1, usually cause amino acid substitutions that increase *p110δ* enzyme activity, such as the *E1021K* mutation [15]. In contrast, *PIK3R* mutation is related to the incidence of APDS2, which prevents the interaction between *p85α* and *p110δ*, causing uncontrolled management of the enzyme subunits [16,17]. Both subtypes are inherited in an autosomal dominant pattern. The literature reports both familial and sporadic cases associated with de novo mutations. Additionally, paternal and maternal gonadal mosaicism has been suggested in relation to APDS1, which helps clarify the confusing inheritance pattern of this disease [18,19]. Most importantly, these genetic results highlight the need for genetic testing for the precise diagnosis and classification of APDS, which is vital for directing personalized treatments.

Impact on Immune System Regulation

Proper immune response control depends much upon the PI3Kδ pathway [5]. B-cell and T-cell activation, proliferation, and regulatory T-cell differentiation (Tregs) [20,21] depend critically on it. Especially vital for antigen receptor-mediated lymphocyte activation is PI3Kδ signaling; it helps immune cell survival and generates vital cytokines [3]. Sustained hyperactivation of this pathway follows gain-of-function mutations in *PIK3CD* and *PIK3R1*, causing several different immune dysregulations [22]. Overactivity in PI3Kδ signaling, for example, allows for lymphocyte growth and survival, thereby causing lymphadenopathy and splenomegaly, characteristic signs of APDS [22]. In addition, this disorder is associated with frequent infections and a weakened immune system defense due to the dysregulation of signaling that inhibits the formation of T-cell memory and the production of B-cell antibodies. The complexity of the condition and the need for specific treatments that can help balance the immune system are underscored by this paradox of hyperactivity, resulting in immune system exhaustion.

Mechanisms Leading to Immunodeficiency and Dysregulation

**Figure 1:**
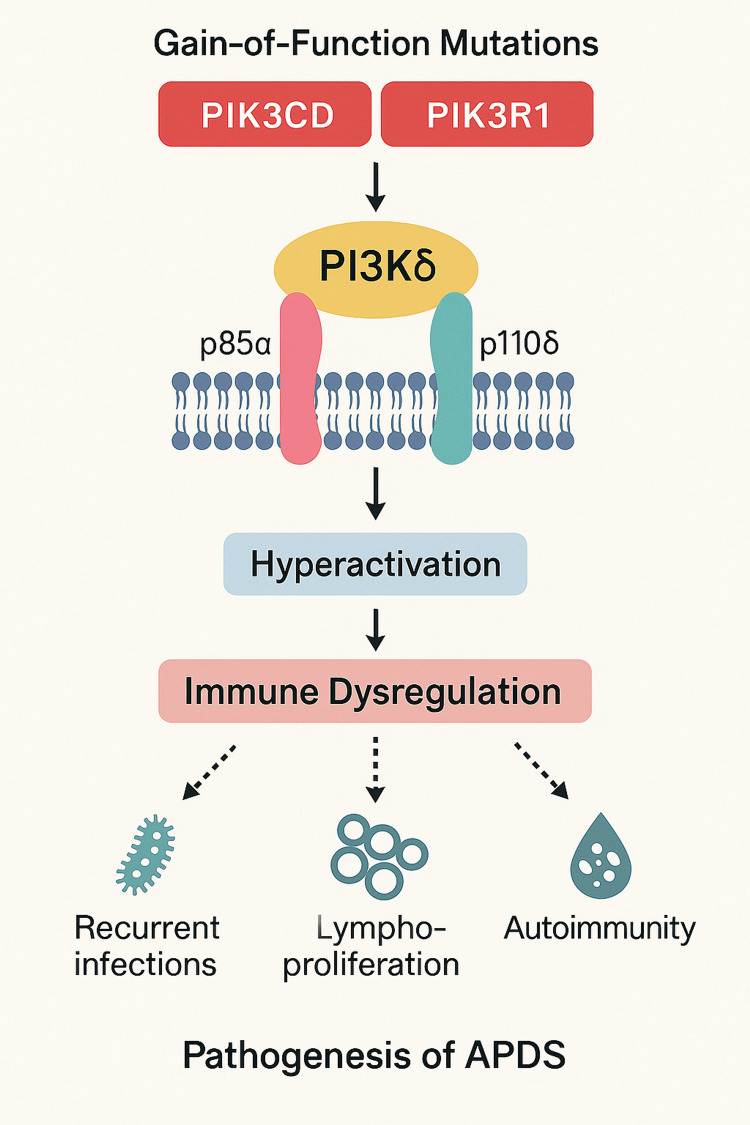
Pathogenesis of Activated PI3 Kinase Delta Syndrome (APDS). Image Credit: Elias A. Alraqibah

The hyperactivation of the PI3Kδ pathway in APDS has significant consequences for immune system equilibrium. One major effect is the disturbance of regular lymphocyte growth and activity [23]. B cells display compromised class-switch recombination, resulting in lower levels of immunoglobulin subclasses and scant vaccine and infection antibody reactions [24,25]. T cells also show decreased activity, including a reduced ability to mount efficient reactions to pathogenic insults. Furthermore, chronic PI3Kδ pathway activation aids in immune dysregulation, which manifests as inflammatory symptoms and autoimmunity. APDS patients, for instance, frequently develop autoimmune cytopenias, symptoms similar to those of inflammatory bowel disease, and other symptoms of immune hyperactivation [26]. These events have a molecular basis, including the direct effects of PI3Kδ hyperactivity and secondary changes in downstream signaling molecules, such as the mTOR pathway and transcription factors controlling immune regulation [14]. Together, these processes account for the complex clinical presentation of APDS, which includes both immunodeficiency and immune regulatory features (Figure 1).

Splice donor site mutations, such as c.1425+1G> (A, C, T) (p.434-475del), lead to exon 11 skipping in the *p85α* subunit, which disrupts its regulatory function. The *p110δ* subunit catalyzes the conversion of phosphatidylinositol-4,5-bisphosphate to phosphatidylinositol-3,4,5-trisphosphate, which anchors signaling proteins like PDK1 and AKT. Mutations in *p110δ* result in the hyperactivation of the AKT/pS6K/mTOR pathway, classifying them as gain-of-function mutations, whereas mutations in *p85α* remove its inhibitory control over p110, increasing PI3K activity. Since the mTOR pathway regulates essential cellular processes, such as growth, metabolism, and immune function, its dysregulation leads to significant immune system alterations in affected individuals [27,28].

Epidemiology and demographics

Global Prevalence of APDS

APDS is a rare primary immunodeficiency disorder, and its exact prevalence has not yet been fully established [13]. The prevalence of APDS across studies has shown its scarcity worldwide [10,11,13], ranging from studies that did not report a prevalence to others that reported only one to two cases per million individuals [11,13]. However, several studies have found that there is a perception that this frequency is an underdiagnosis owing to its clinical overlap with symptoms of several other immunodeficiencies and other immune dysregulation syndromes [10,12,13]. Developments in genetic testing and the rise of whole-exome sequencing have helped identify more cases in recent years, indicating that APDS's actual frequency might be higher than that currently published [13]. Notwithstanding its infrequency, it is acknowledged as a major cause of morbidity associated with immune dysregulation in affected individuals, pointing to the need for genetic counseling and clinical management [26]. In regions where access to genetic testing is limited, ongoing research is expected to elucidate the global impact of APDS.

Regional and Cohort-Specific Variations

The rate of APDS cases has been reported in different regions, indicating some regional variations that describe and identify its existence [12]. Studies have shed light on instances in Europe, North America, the Middle East, and Asia, with different clinical presentations depending on the population investigated. Cohorts from Western countries, for instance, usually have earlier access to diagnostic equipment and, therefore, more regular identification of the disease [13]. In regions with constrained health resources, underdiagnosis is more common due to the unavailability of diagnostic tools, such as genetic testing or immunologic evaluation [13]. This provides evidence that certain genetic pools may have higher pathogenic mutation levels, urging more epidemiological investigations to investigate these patterns. Understanding local variations in clinical presentation, disease burden, and treatment responses depends on cohort-specific investigations, which, in the end, inform customized patient care methods.

Age of Onset and Gender Differences

Most APDS cases are identified in the first two decades of life, and the condition usually appears in childhood or adolescence [10,13,29]. Bloomfield et al. presented eight case reports of patients with APDS and found the median age at diagnosis of APDS to be 16 years (range 2-30 years) [27]. Another study by Tessarin et al. showed a median age of 12.9 years and a range of 2.2 to 43.2 years [28]. However, variability exists in the age of symptom onset; some people may show early signs of the disorder in infancy, but others may remain undiagnosed until adulthood [30,31]. APDS is often indicated by the early onset of multiple infections, lymphoproliferation, or autoimmune disorders; therefore, more studies are required [13]. Both men and women seem to suffer equally from the frequency and harshness of APDS; therefore, sex variations have not been definitively determined. Moreover, the clinical presentation of this disorder may be changed and affected by different genetic, hormonal, or environmental factors, including the age of diagnosis and symptom progression in patients [30,31]. Clinicians must observe these demographic trends to identify at-risk individuals and apply early treatment.

Clinical features of APDS

Immunological Manifestations

Immune abnormalities, ranging from hyperactivation of the PI3Kδ pathway, define APDS [13]. These changes primarily influence lymphocyte development, signaling, and activity. Hyperactivation of PI3Kδ in B cells impairs class-switch recombination, thereby decreasing IgG and IgA levels, as well as the overall immunoglobulin class [32]. Consequently, individuals frequently experience hypogammaglobulinemia or dysgammaglobulinemia, rendering them more vulnerable to infections. Similarly, T-cell function is impaired, with fewer functional memory T cells being created and weakened cytotoxic activity [12]. Tregs, vital for preserving immune tolerance, are also compromised, further contributing to immune dysregulation and autoimmunity [33]. Moreover, reduced natural killer (NK) cell activity worsens the immune reaction to viral agents [7]. These immune signs emphasize the difficulty in detecting and treating APDS due to its dual nature as an immune dysregulation disorder and a primary immunodeficiency, a uniquely complex condition [13].

Recurrent Infections and Lymphoproliferation

One of the defining characteristics of APDS is repeated infections, which are sometimes serious and difficult to control. These are typical viral illnesses that often affect the respiratory system. The weakened antibody-mediated immune reaction helps to make encapsulated bacteria, including *Streptococcus pneumoniae* and *Haemophilus influenzae*, the most common perpetrators [10]. Viral infections, especially those induced by herpesviruses, including Epstein-Barr virus (EBV) and cytomegalovirus (CMV), are also common and can cause long-term and severe illness [10]. Repeated respiratory infections can lead to bronchiectasis, increasing long-term morbidity [34]. In addition, skin abscesses are common in patients with Staphylococcus aureus infection [35,36]; however, invasive bacterial infections are rare [35], and these infections are mostly observed in patients with humoral deficiencies [37].

Lymphoproliferation, shown as lymphadenopathy, splenomegaly, and hepatomegaly, is another well-known clinical sign of APDS, reported in 75% of APDS1 and 89% of APDS2 patients [35,36]. Uncontrolled lymphocyte proliferation from the hyperactive PI3Kδ pathway causes these symptoms [38]. Not only is it a diagnostic indicator, but it also causes significant clinical anxiety, as it may predispose patients to the development of lymphoma, especially non-Hodgkin lymphoma [38]. Both APDS subtypes are at a higher risk of developing different types of B-cell lymphoma, particularly classical Hodgkin lymphoma, diffuse large B-cell lymphoma, and marginal zone B-cell lymphoma [35,39,40].

Autoimmune and Inflammatory Complications and Developmental Delay

In addition to immunodeficiency, APDS is associated with several autoimmune and inflammatory issues, showing dysregulated immune activation due to PI3Kδ hyperactivity [7]. Autoimmune cytopenias, including autoimmune hemolytic anemia, immune thrombocytopenia (ITP), and autoimmune neutropenia, are common and frequently observed early in the disease progression. Inflammatory disorders similar to systemic autoimmune diseases, including vasculitis or lupus-like syndromes, are also possible [41].

Gastrointestinal inflammation can resemble inflammatory bowel disease (IBD) in patients with APDS, which is another serious problem. These signs of chronic diarrhea, stomach discomfort, and malabsorption have a serious impact on the QoL [42]. These autoimmune and inflammatory phenomena indicate the complexity of this condition, combining reduced immunity and an excessive immune response. Tailoring treatment strategies that frequently combine immunosuppressive drugs, targeted therapies, and supportive care to address the numerous clinical issues posed by APDS depends on a thorough understanding of these problems. Additionally, another disorder associated with this condition is neurodevelopmental abnormalities, including speech delay or global developmental delays, which are more commonly reported in patients with APDS2 [11].

Diagnosis of APDS

Genetic Testing and Diagnostic Biomarkers

A definitive diagnosis of APDS relies on DNA testing to identify pathogenic variations in the *PIK3CD* or *PIK3R1* genes [10,12]. Whole-exome sequencing (WES) or targeted gene panels for primary immunodeficiencies have become indispensable for diagnosing APDS in patients with recurrent infections, lymphoproliferation, and immune dysregulation [13]. In addition to verifying the diagnosis, genetic testing differentiates between the two subtypes of APDS: APDS1, caused by gain-of-function *PIK3CD* mutations, and APDS2, caused by *PIK3R1* mutations [8]. Early discovery of these mutations is vital, as it allows the early start of tailored treatments, such as PI3Kδ inhibitors.

Apart from genetic testing, the number of diagnostic biomarkers is increasing. Frequently observed in patients with APDS, increased serum IgM levels might provide a starting point [43,44]. Flow cytometry is another useful tool that exposes distinctive immunologic anomalies, including lower numbers of naive T cells, poor memory B cells, and elevated transitional B cells [45]. Further clues into the functional effects of the mutations might be provided by other biomarkers, including hyperphosphorylation of AKT (a downstream target of PI3Kδ) [13]. These diagnostic tools help doctors make accurate diagnoses and better understand the molecular and immunological aspects of the condition.

Differentiation From Other Immunodeficiencies

APDS exhibits clinical and immunological characteristics compared to other primary immunodeficiency disorders, making it a vital part of the diagnosis. Symptoms of conditions, including common variable immunodeficiency (CVID), hyper-IgM syndrome, and other combined immunodeficiencies, usually overlap to include recurrent infections, lymphoproliferation, and autoimmune problems [46]. Still, some characteristics of APDS can assist in differentiating it from these disorders [13]. For example, lymphadenopathy, splenomegaly, and raised serum IgM levels are more typical of APDS. Moreover, while CVID generally appears later in life, APDS usually starts earlier, sometimes even in childhood or adolescence [10,12,13].

As it identifies the particular gain-of-function mutations in *PIK3CD* or *PIK3R1* that define APDS, molecular diagnosis via genetic testing is the most dependable way of differentiation [10,12]. Functional experiments, including PI3Kδ overactivation research, can also show abnormal activation of the PI3K-AKT-mTOR pathway [13,46], further justifying the diagnosis. Understanding these differences is key to guaranteeing that patients receive the proper treatment since the therapy for APDS varies quite from that for other immunodeficiencies.

Challenges in Early Diagnosis

Early detection of APDS is still difficult, given its clinical heterogeneity and overlap with other immunological disorders, even if genetic testing technologies have improved [13]. With symptoms sometimes wrongfully imputed to more prevalent illnesses like autoimmune diseases or recurrent infections, many patients suffer a late diagnosis [10]. If left untreated, this diagnostic delay can lead to serious consequences such as permanent organ damage, chronic lung disease, and the development of cancers like lymphoma.

Another difficulty is the variation in the symptom presentation. Though some patients show traditional signs of immunodeficiency, such as frequent respiratory infections, others could have autoimmunity or inflammatory symptoms primarily, therefore complicating the diagnosis [13]. Furthermore, compounding the problem of late diagnosis is the restricted availability of genetic testing and sophisticated immunological evaluations, especially in poor settings [10].

Therapeutic approaches

Evolution of Targeted Therapies

Over the last decade, the APDS treatment scenario has changed considerably from broad immunosuppression and supportive means to molecularly targeted therapies [13,47-49]. Historically, treatment for APDS has mostly concentrated on managing the symptoms and problems of the illness, including infections, autoimmunity, and lymphoproliferation, using broad-spectrum antibiotics, immunoglobulin substitution therapy, and corticosteroids. However, these therapies provide only moderate control and do not address the fundamental genetic and molecular dysfunctions that fuel the disease [47].

The discovery of gain-of-function mutations in *PIK3CD* and *PIK3R1* heralded a new era of targeted therapies. These therapies seek to address the underlying cause of APDS by directly suppressing the overactive PI3Kδ pathway, thereby offering more accurate and efficient disease management [23]. Clinical trials of PI3Kδ inhibitors have yielded promising results, demonstrating significant enhancements in immune function, reductions in lymphoproliferation, and stabilization of autoimmune disorders [23]. The arrival of these treatments has changed the way APDS is managed. Hence, patients have hope for an improved QoL and greater disease control.

Role of PI3Kδ Inhibitors (e.g., Leniolisib, Duvelisib)

Due to their capacity to selectively target the hyperactive PI3Kδ pathway, PI3Kδ inhibitors like leniolisib and duvelisib have become fundamental treatments for APDS [50,51]. Leniolisib, a selective PI3Kδ inhibitor, has shown strong activity in APDS patients in reducing lymphoproliferation, boosting the immune system, and relieving autoimmune symptoms. Depending on clinical studies, leniolisib not only reduces spleen size and lymphadenopathy but also enhances important immune regulation markers, including normalization of immunoglobulin levels and recovery of T-cell activity [15,50,52].

Another PI3Kδ inhibitor, duvelisib, has helped to control both autoimmune symptoms and lymphoproliferative features of APDS [50,53]. Originally created for blood cancers, its use in APDS has been investigated since its theory dampens overactive immune signals [50,53]. These inhibitors, like all targeted treatments, have possible adverse effects, including raised infection susceptibility and cytopenias, thus requiring close monitoring throughout therapy [50]. The advent of PI3Kδ inhibitors has transformed APDS treatment, offering specific and potent choices previously not available [53]. These treatments emphasize the need for accurate medicine in tackling uncommon genetic conditions like APDS.

Role of mTOR Inhibitor

Sirolimus (rapamycin) is another medication that inhibits mTOR, which is involved in T-cell metabolism and immune regulation [12]. This medication has been found to be useful in the reduction of hepatosplenomegaly and lymphadenopathy, restoring T-cell proliferation, and managing non-neoplastic lymphoproliferation; however, it has a less satisfactory response in the management of cytopenia and gastrointestinal symptoms [12].

Immune-Modulatory Therapies and Supportive Care

In particular, for treating autoimmune and inflammatory problems, immunomodulatory therapies, in addition to targeted therapies, continue to be a key part of APDS management [8,13]. Corticosteroids are frequently used to manage episodes of acute inflammation, such as vasculitis and autoimmune hemolytic anemia. However, they have significant side effects that restrict their long-term use [54-56]. Other immunosuppressive drugs, such as rituximab, have been employed to treat intense lymphoproliferation and unresponsive autoimmune cytopenias with relatively good clinical results [54-56].

Supportive care plays a crucial role in improving the QoL of individuals with APDS. Immunoglobulin replacement therapy is often used to treat hypogammaglobulinemia and reduce the chances of repeated infections [13]. This supportive care is critical in patients with compromised T-cell or NK cell function, where the application of antibiotics and antifungal medications is critical to avoid the incidence of serious infections [13]. Vaccination is another approach used to protect against particular pathogens. However, its administration depends on the level of immune dysfunction in patients. Together, these immune-modulating medications and care approaches support targeted treatments for thorough APDS disease management.

Hematopoietic Stem Cell Transplantation (HSCT)

HSCT is a possible treatment for those with advanced or obstinate APDS [57]. Replacing a patient's faulty immune system with donor-derived stem cells effectively addresses the underlying genetic and molecular problems [58,59]. Patients with aggressive immune dysregulation, life-threatening lymphoproliferation, or cancer that do not respond to other therapies especially benefit from this method [58]. Although HSCT provides a possibility of cure, it comes with major hazards, such as graft-versus-host disease (GVHD), infections, and transplant-related death [60,61]. Patient age, disease severity, and the presence of a good donor all influence the success of HSCT [62]. Although HSCT is still reserved for meticulously selected cases in which the advantages exceed the risks, advances in conditioning regimens and supportive care have improved the outcomes of HSCT for APDS [50].

Outcomes and prognosis

Response of the Patients to the Treatment and the Long-Term Outcomes

The outcomes of patients diagnosed with APDS have greatly improved after the introduction of these treatments, including PI3Kδ inhibitors. Many treatments result in significant decreases in lymphoproliferation, better management of autoimmune signs, fewer recurrent infections, and relief for patients. Leniolisib, for example, has shown continuous efficacy in clinical studies, including decreases in spleen and lymph node size and increased immune response [57]. Rituximab and other treatments, such as duvelisib and immunomodulators, have also proven effective in controlling serious lymphoproliferative and autoimmune disorders [8].

The development of APDS depends on the age at diagnosis, the extent of signs of the disease, and the promptness of treatment [10]. Early recognition of a problem and initiation of therapy are essential in lowering irreversible damage, such as organ dysfunction or chronic lung damage caused by continuous inflammation or infections [13]. Current treatment strategies provide patients with significant relief and control of the condition. However, they do not cure the disease [11]. Therefore, to ensure the best outcomes for the patient, lifetime monitoring and continual treatment changes according to disease progression are frequently needed.

Risk of Treatment-Associated Complications

Although not risk-free, targeted therapies and immunomodulatory medications have transformed APDS treatment. For example, by virtue of their immunosuppressive qualities, PI3Kδ inhibitors could raise susceptibility to infections [43,50]. Careful monitoring and prophylactic measures are necessary for patients receiving these treatments due to the risk of opportunistic infections, including viral or fungal reactivations [6]. Furthermore, in some individuals, these drugs may be limited by other side effects, including cytopenia, hepatitis, and gastrointestinal issues [16]. The risk of complications is significantly higher in patients who undergo HSCT. Common issues include transplant rejection, GVHD, and treatment side effects. Given the extreme immunosuppression required for surgery, infections remain a major concern during the post-transplantation phase [50,62].

QoL and Prognostic Indicators

The chronicity of the illness and its related side effects considerably affect the QoL of individuals living with APDS. Patients usually grapple with several issues, such as frequent infections, fatigue, lymphoproliferation, and autoimmune diseases, all of which can affect their social, emotional, and physical health. Further aggravating the difficulties faced by these patients are the long-term effects of the disease, including bronchiectasis, organomegaly, and cancer [8,13]. The constant need for hospital stays, continuous therapies, and possible side effects all contribute to a low QoL. Despite these difficulties, several elements have been found to predict better results. Early detection and timely introduction of personalized remedies are vital for reducing damage and retaining organ function [13]. Regular monitoring and supportive treatment, including immunoglobulin replacement therapy, prophylactic antibiotics, and physiotherapy for lung health, also increase the QoL [8]. Therefore, the development of new treatment options and the use of possible gene-editing techniques are expected to further improve this field.

## Conclusions

APDS represents a rare yet significant primary immunodeficiency disorder driven by gain-of-function mutations in the *PIK3CD *or *PIK3R1* genes, leading to hyperactivation of the PI3Kδ pathway. This review has highlighted the complex interplay between immunodeficiency and immune dysregulation in APDS, characterized by recurrent infections, lymphoproliferation, autoimmune complications, and inflammatory manifestations. Advances in genetic sequencing and biomarker analysis have improved diagnostic accuracy, enabling earlier intervention and tailored therapeutic strategies. The emergence of PI3Kδ inhibitors, such as leniolisib and duvelisib, has revolutionized treatment by directly targeting the underlying molecular defect, demonstrating efficacy in reducing lymphoproliferation and stabilizing immune function. Additionally, mTOR inhibitors, immunoglobulin replacement therapy, and HSCT offer alternative approaches for severe or refractory cases. Despite these advancements, challenges remain, including delayed diagnosis, treatment-related complications, and variability in clinical presentation.

Future research should focus on optimizing long-term outcomes, exploring novel targeted therapies, and investigating gene-editing technologies as potential curative interventions. A multidisciplinary approach involving immunologists, geneticists, and hematologists is essential to address the diverse clinical manifestations of APDS and improve patients' QoL. As our understanding of APDS continues to evolve, further discoveries in precision medicine hold promise for more effective and individualized treatment strategies. This review underscores the importance of continued research and collaborative efforts to refine diagnostic and therapeutic paradigms, ultimately enhancing care for individuals affected by this complex disorder.
